# Management of Cardiovascular Diseases in Chronic Hemodialysis Patients

**DOI:** 10.31083/j.rcm2407185

**Published:** 2023-06-29

**Authors:** Zhen Zhang, Yaqiong Wang

**Affiliations:** ^1^Department of Nephrology, Zhongshan Hospital, Fudan University, 200032 Shanghai, China; ^2^Shanghai Medical Center for Kidney Disease, Shanghai Municipal Health Commission, 200032 Shanghai, China; ^3^Shanghai Institute of Kidney and Dialysis, 200032 Shanghai, China; ^4^Hemodialysis Quality Control Center of Shanghai, Shanghai Medical Quality Control Management Center, 200032 Shanghai, China

**Keywords:** hemodialysis, end-stage kidney disease, cardiovascular disease

## Abstract

Hemodialysis (HD) is the main treatment modality for patients with end-stage kidney disease. Cardiovascular diseases (CVD) are highly prevalent in HD patients and are the leading cause of death in this population, with the mortality from CVD approximately 20 times higher than that of the general population. Traditional and non-traditional cardiovascular risk factors accelerate progression of CVD and exacerbate the prognosis in HD patients. This review provides a brief overview of the characteristics of CVD in HD patients, and a description of advances in its management.

## 1. Epidemiology of Cardiovascular Diseases (CVD) in Hemodialysis (HD) Patients

CVD is the leading cause of death in patients undergoing HD. Although patients with end-stage kidney disease (ESKD) tend to have hypertension and diabetes mellitus (DM), which are major risk factors for the progression of CVD, studies have shown that ESKD is still an independent risk factor for CVD, distinct from hypertension and DM [1, 2]. CVD in HD patients is mainly manifested as left ventricular hypertrophy (LVH), coronary artery disease (CAD), heart failure (HF), arrhythmias, and sudden death. More than 50% of HD patients are reported to have CVD, and the relative risk of death from CVD events in HD patients is 20 times higher than that in the general population.

Globally, 70–90% of HD patients have hypertension [3] and 60–80% develop LVH [4, 5], mostly due to eccentric ventricular remodeling induced by increased volume overload (VO) and concentric remodeling induced by increased afterload (high peripheral resistance). Other factors include high cardiac output induced by anemia and arteriovenous fistula, altered central arterial compliance, and dysregulation of neurohormonal systems such as the Renin-Angiotensin-Aldosterone System (RAAS) [6]. Studies have shown that LVH is strongly associated with cardiovascular mortality in patients with chronic kidney disease (CKD) and that the incidence and severity of LVH progressively increase with the progression of CKD [7].

The European Society of Cardiology (ESC) Guidelines for the diagnosis and treatment of acute and chronic HF classify heart failure into HF with reduced ejection fraction (HFrEF) (LVEF ≤40%), HF with preserved ejection fraction (HFpEF) (LVEF ≥50%), and HF with mid-range ejection fraction (LVEF 41–49%) [8]. It is reported that about 44% of HD patients have HF, 10% have HFpEF, and 13% have HFrEF [9].

CAD is common in patients with CKD, especially in those on HD. The United States Renal Data System (USRDS) report showed that the annual incidence of myocardial infarction and/or angina pectoris in dialysis patients is about 10%. Charytan *et al*. [10] found that in HD patients without angina pectoris, around 40% (28 of 67) had ≥50% stenosis in at least one major coronary artery, and 19 patients had severe coronary stenosis. Mortality rates are high in HD patients who develop a myocardial infarction. According to the USRDS data, after an acute myocardial infarction (AMI), the in-hospital mortality rate was 18.8%, and the unadjusted 2-year cumulative probability of death after AMI admission was 71.5% [11].

It is currently estimated that 25% of all-cause deaths among dialysis patients are caused by sudden cardiac death (SCD) [12]. Arrhythmias and sudden cardiac arrest (SCA) are important causes of SCD. The incidence of SCA in dialysis is 4.5–7.0/100,000 dialysis sessions [13, 14]. Despite the low incidence, the outcome of SCA in dialysis is poor. Karnik *et al*. [13] observed that only 40% of patients with SCA were successfully resuscitated and remained alive after 2 days, 60% died within 48 hours after the cardiac arrest, and 13% died in the HD unit. In ambulatory patients, the most frequent cause of SCD is ventricular tachyarrhythmias, with ventricular fibrillation (VF) being the most frequent ventricular tachyarrhythmia [15]. By monitoring 75 HD patients using a wearable cardioverter-defibrillator, it was found that 78.6% of SCA events were due to ventricular tachycardia (VT) or VF, while asystole accounted for 21.4% [16]. In addition, studies have shown that SCD is related to the timing of HD, and occurs during two time intervals, one at the end of a longer dialysis run, and the other during the initial dialysis period [17, 18]. As expected, there is a significant correlation between pre-dialysis hyperkalemia and SCD. Patients are at a higher risk of conduction disorders when serum potassium is >5.0 mmol/L. A higher risk for ventricular arrhythmias was associated with a potassium <4.0 mmol/L [19]. In summary, during the dialysis interval, HD patients undergo a relatively rapid transition from mild hypokalemia or normokalemia to hyperkalemia and metabolic acidosis, both of which lead to cardiac electrophysiological instability, resulting in life-threatening arrhythmias.

## 2. Non-Traditional Risk Factors for CVD in HD Patients

In addition to traditional CVD risk factors such as hypertension, dyslipidemia, and smoking, non-traditional risk factors in HD patients also play an important role in the development of cardiovascular disorders. More effective control of risk factors may contribute to improved survival in HD patients.

### 2.1 VO and Dialysis-Induce Systemic Stress

VO is directly linked to cardiac remodeling, with recurrent stretching of cardiac chambers [20]. VO is strongly associated with cardiovascular morbidity and mortality. Patients with higher interdialytic weight gains (IDWG) had higher pre-dialysis blood pressure and a higher risk of all-cause and CV mortality [21]. Studies with more objective volume assessment using bioimpedance analysis found that baseline VO and chronic exposure to VO were associated with death in HD patients [22, 23, 24].

**Fig. 1.**
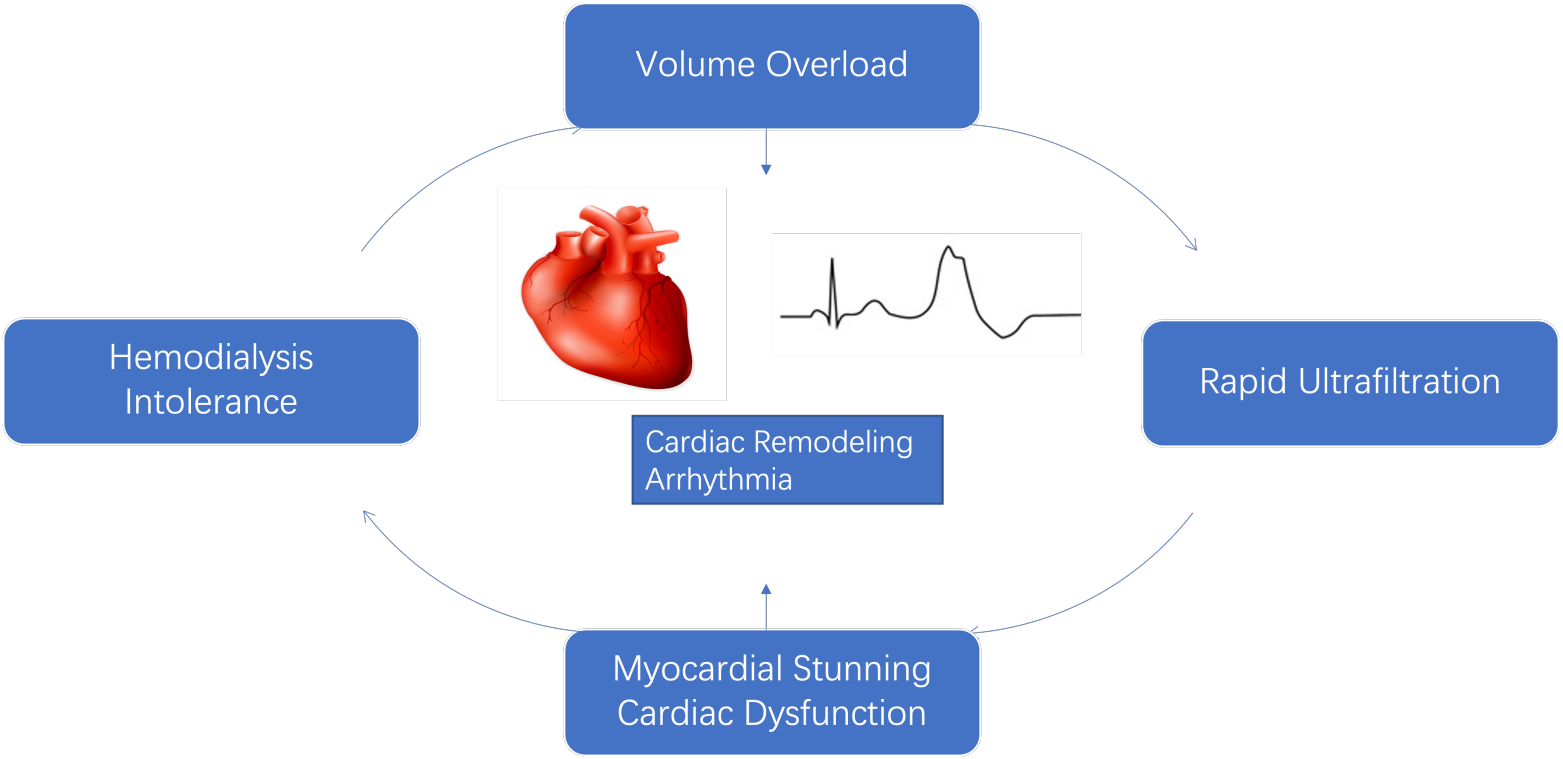
**Potential impact of volume overload, increased ultrafiltration rate and adverse cardiac outcome**.

“Standard” 4-hour, thrice-weekly HD has been the major treatment schedule in most dialysis centers for decades. Unlike continuous urine production by the kidneys, HD is an intermittent therapy that rapidly removes fluid during each session. In anuric HD patients, the fluid volume accumulated between HD sessions almost equals the prescribed ultrafiltration volume. Observational data consistently demonstrated a strong association between high ultrafiltration rate (UFR) and greater mortality, with a threshold around 10–13 mL/kg/hr [25, 26, 27]. Rapid fluid removal from intravascular compartment during HD, if not compensated by plasma refilling and proper baroreflex, would impose hemodynamic stress and cause intradialytic hypotension (IDH), resulting in intolerance to HD sessions or inaccurate adjustment of dry weight, thus aggravating VO. High UFR could cause end-organ hypoperfusion even without IDH. Studies have demonstrated that HD could induce global and segmental myocardial ischemia and myocardial regional wall motion abnormalities (RWMAs) [28, 29, 30, 31]. Repetitive myocardial injury would accelerate cardiac remodeling and compromise HD tolerance. Patients with HD-induced RWMAs have more premature ventricular complexes [32], decreased ejection fraction [28] and higher mortality [33] (Fig. 1).

Similar adverse effects also occur in other end-organs, including the gut, skeletal muscle, and brain, which may in turn accentuate HD intolerance and systemic inflammation. VO may induce inflammation by damaging the integrity of the bowel wall and the translocation of endotoxin [34]. Inflammation, by increasing capillary permeability and causing hypoalbuminemia, might induce interstitial fluid retention, compromise plasma refilling and ultrafiltration intolerance [35].

This vicious cycle derived from the unphysiologic nature of intermittent HD was summarized in the term “dialysis-induced systemic stress (DISS)”, emphasizing the imperfection and flaws of current HD therapy [36, 37]. The term DISS encompasses both hemodynamic and non-hemodynamic stress factors.

### 2.2 Uremic Toxin Retention

As kidney function decreases, uremic toxins accumulate and become biologically active, exerting adverse effects on the cardiovascular system.

Despite the introduction of high-flux dialysis and convective therapy, the removal of protein-bound solutes remains limited. The two iconic protein-bound toxins are p-cresol and indoxyl sulfate (IS). Studies have shown that p-cresol accumulation in CKD patients is closely associated with cardiovascular risk in CKD and is predictive of mortality [38]. *In vitro* studies have shown that p-cresol causes endothelial cell dysfunction *via* a toxic mechanism mediated by Rho kinase activity [39]. Another protein-bound uremic toxin, IS, is derived from tryptophan metabolism and is highly bound to albumin. IS has pro-oxidant and pro-inflammatory effects, triggers an immune response, accelerates CKD progression, and increases the occurrence of CVD events [40]. IS is also a potential CKD-associated pro-thrombotic uremic toxin, inducing tissue factor expression in vascular smooth muscle cells, and increasing the risk of pro-thrombotic properties after vascular intervention in a tissue factor-dependent manner [41].

### 2.3 Oxidative Stress, Endothelial Cell Dysfunction

The kidney is one of the most important sources of antioxidant enzymes, and decreased kidney function leads to an increase in pro-oxidant substances. Oxidative stress is common in ESKD, which accelerates renal injury by promoting renal ischemia, inducing apoptosis, and stimulating inflammatory responses. Increased levels of asymmetric dimethylarginine (ADMA) in ESKD lead to endothelial dysfunction by inhibiting endothelial cell NO synthase. ADMA levels in ESKD patients are closely associated with endothelial dysfunction as well as cardiovascular events [42]. The depletion of antioxidants and accumulation of oxidation products during HD also result in excessive oxidative stress. In addition, the HD procedure itself promotes the production and accumulation of oxidative products by activating platelets, complement and polymorphonuclear cells, and significantly increasing plasma ROS levels after the HD session [43].

### 2.4 Chronic Kidney Disease-Mineral and Bone Disorder (CKD-MBD) and Cardiovascular Calcification

Cardiovascular calcification is a well-established and widely acknowledged cardiovascular risk factor in ESKD and HD patients. Vascular calcification involves the trans-differentiation of vascular smooth muscle cells into osteoblast-like cells that induce a phenotypic shift by upregulating the growth of osteochondrogenic markers, and ultimately initiating the local mineralization process [44]. In dialysis patients, cardiac valve calcification (CVC) in CKD-MBD increases the risk of arrhythmias, SCD, stroke, and mortality. Aortic stenosis is a common consequence of the calcification process, increasing cardiac afterload and further contributing to LVH. A meta-analysis confirmed that the higher the degree of CVC, the higher the mortality in dialysis patients, with CVC increasing cardiovascular mortality by 181% and all-cause mortality by 73% [45]. In addition, studies suggest that CKD-MBD biomarkers such as fetuin-A, osteoprotegerin, and osteopontin are associated with vascular calcification in HD patients. Fetuin-A is a hepatocyte-derived glycoprotein and a potent inhibitor of systemic calcification by facilitating clearance of mineral crystals deposited in the tissues. Compared with healthy controls, plasma fetuin-A concentrations are lower in HD patients, and are associated with vascular calcification and arterial stiffness, as well as increased all-cause and cardiovascular mortality [46].

### 2.5 Fibroblast Growth Factor-23 (FGF-23) and Klotho

Fibroblast growth factor-23 (FGF-23), a protein secreted by osteoclasts and osteoblasts, works with parathyroid hormone (PTH) in the regulation of phosphate excretion by interacting with the FGF receptor. FGF-23 requires the co-receptor α-Klotho for its physiological activity. FGF-23 reduces blood phosphorus levels in a Klotho-dependent manner by inhibiting 1,25-hydroxyvitamin D and PTH synthesis [47]. It was found that elevated plasma FGF-23 levels were independently associated with rapid CKD progression and CVDs in ESKD patients. FGF-23 caused pathological hypertrophy of isolated rat cardiomyocytes *via* FGF receptor-dependent activation of the calcineurin-NFAT signaling pathway, but this effect was independent of Klotho [48]. A growing body of evidence from animal experiments suggests that Klotho deficiency leads to vascular calcification, myocardial fibrosis and myocardial hypertrophy in patients with CKD [49]. In addition, reduced Klotho production makes the kidney more susceptible to injury and exacerbates uremic cardiomyopathy and vascular calcification.

### 2.6 Gut Microbes as a Potential Source of Uremic Toxins

The gut microbiome (GM) is now considered to be a metabolically active endogenous organ. Repeated ultrafiltration or fluid removal during HD sessions causes intestinal ischemia, which alters the integrity of the intestinal wall and disrupts the intestinal barrier, resulting in the translocation of bacteria and endotoxins in the circulatory system. Intestinal microecological dysregulation stimulates pro-inflammatory cytokine production, foam cell formation, and oxidative stress, which in turn increases the inflammatory state. Studies of GM alteration in CKD patients revealed that the proportion of microbiota (*Bifidobacterium spp.* and *Enterobacteriaceae*) is significantly reduced. Changes in the microbiota produce excess uremic toxins such as p-cresol sulfate, IS and trimethylamine nitrogen oxide, and these enteric-derived uremic toxins promote the progression of CKD and CVD [50].

## 3. HD Optimization for Prevention and Management of CVDs

In dialysis-dependent ESKD patients, the kidneys are incapable of producing sufficient urine to regulate salt and water balance in the body. HD removes fluid and solutes via diffusion and convection. In this section, we briefly describe the current understanding of the optimization of HD that may substantially improve CKD patients’ CV outcomes.

### 3.1 HD Schedule and Volume Control

The first step to achieving desirable volume control is to accurately assess the volume status of the patients and probe the target post-dialysis weight or “dry weight”. There is no gold standard for dry weight as the assessment methods are still hight subjective, largely depending on clinical judgment by the dialysis staff, taking into account edema, blood pressure, heart rate, HD tolerance and cardiac biomarkers. The recent application of more objective methods such as bioimpedance analysis is promising, but need to be tested and validated in larger populations [51].

As previously mentioned, aggressive ultrafiltration damages the cardiovascular system and leads to CVD. Increasing total HD time, by increasing the frequency, or prolonging the duration of each HD session, may attenuate the shortcomings of the conventional schedule and improve volume control, as well as solute removal. Two Frequent Hemodialysis Network (FHN) trials, daily and nocturnal, were conducted to assess the benefits of frequent HD compared with conventional HD [52, 53]. In the FHN trials, HD performed 6 days per week was associated with improvements in mortality or 12-month change in left ventricular mass, and mortality or 12-month change in self-reported physical health. However, these benefits were not observed in nocturnal HD performed six times per week. Frequent nocturnal HD may improve blood pressure control, LVH, phosphate control, and reduce dialysis-induced myocardial stunning [54, 55].

Contrary to frequent HD sessions, incremental HD, a less intensive HD modality with gradual dose increase from once- or twice-a-week to thrice-a-week, has been proposed to preserve residual kidney function (RKF). Preservation of RKF and intradialytic urine volume with incremental HD may provide a more patient-centered treatment [56]. RKF is associated with better volume control [57]. More importantly, patients with RKF experienced other advantages beyond volume compared with oligo-anuric patients, including better quality of life and anemia status, lower C-reactive protein (CRP) levels and erythropoiesis-stimulating agents (ESA) requirements, and ultimately, lower mortality [58, 59, 60].

### 3.2 Implementation of Convective Therapy

Convective therapy was expected to improve the prognosis of dialysis patients through greater and wider clearance of uremic toxins. The HEMO study demonstrated that increasing small molecule solutes (e.g., urea) alone would not improve patient prognosis [61].

Hemodiafiltration (HDF) is a mode of dialysis that combines diffusion and convection to achieve greater removal of solutes in a wide spectrum of molecular weights that includes small solutes and conventional middle molecules. The ESHOL study found that HDF had a lower all-cause and CV mortality when compared with high-flux HD [62]. However, in the convective transport study (CONTRAST), a difference in all-cause mortality vs. low-flux HD was only seen in post hoc analyses of patients with a convective volume >18 L [63]. Similarly, the post-hoc analyses of the Turkish (HDF vs. high-flux HD) study showed a difference in CV mortality only in patients with a convective volume >17.4 L [64]. In addition, the French Convective versus Hemodialysis in Elderly study did not find a significant benefit of HDF in all-cause and CV mortality [65]. The pooled individual analyses of these randomized controlled trials (RCTs) indicated that HDF reduces the risk of mortality in ESKD patients in a convective-volume-dependent fashion [66]. However, these findings, stratified by delivered convection volume, should be considered observational as the included trials were not designed to evaluate convection volumes, since high convection volumes can only be achieved in patients with sufficient blood flow, who tend to have fewer comorbidities such as diabetes and fewer vascular comorbidities.

### 3.3 Dialysate Temperature 

It was hypothesized that lowering dialysate temperature can increase peripheral vascular resistance, thus reducing the risk of IDH and preserving myocardial perfusion. A study suggested an individualized cool HD abrogates myocardial stunning and stabilizes hemodynamics [67]. Data from other studies indicated that the potential benefits of cool dialysis in maintaining blood pressure comes at the cost of more frequent discomfort, such as shivering or cramps [68, 69]. Unfortunately, the latest Personalised cooler dialysate for patients receiving maintenance hemodialysis (MyTEMP) trial, which included 15,413 patients, found that cool dialysis did not reduce the risk of major cardiovascular events after a 4 year follow-up [70].

### 3.4 Dialysate Composition

For HD patients, especially those with complete loss of kidney function, dialysis is the most important measure of electrolyte removal. The appropriate dialysate composition is crucial in regulating the electrolyte balance in the body. In this section, we focus on two key electrolytes: sodium and potassium.

### 3.5 Sodium

Currently, most dialysis centers adopt a dialysate sodium concentration (${\mathrm{Na}}_{\text{d}}$) of around 140 mEq/L. Studies have demonstrated that either a high or low ${\mathrm{Na}}_{\text{d}}$ has potential clinical risks and benefits. Higher ${\mathrm{Na}}_{\text{d}}$ usually improves intradialytic hemodynamic stability and HD tolerance at the expense of volume expansion, high blood pressure, and more IDWG. On the other hand, lower ${\mathrm{Na}}_{\text{d}}$ is associated with lower IDWG and blood pressure, but a higher incidence of IDH and intradialytic discomfort. According to the Dialysis Outcome and Practice Patterns Study (DOPPS) data, higher ${\mathrm{Na}}_{\text{d}}$ was associated with lower mortality in patients with a lower pre-dialysis serum sodium concentration [71]. Currently, there is no evidence supporting an optimal fixed sodium dialysate. Therefore, the choice of sodium concentration should be individualized, taking into account the pre-dialysis serum sodium level, HD tolerance, and volume status.

### 3.6 Potassium

Hyperkalemia is associated with poor outcomes in patients undergoing HD [72, 73]. HD can efficiently reduce serum potassium concentration ($K_{\text{s}}$), but post-dialysis hypokalemia is associated with an increased incidence of ventricular arrhythmias and death [19, 74]. Since high or low serum potassium levels can result in adverse effects, a fixed dialysate potassium concentration ($K_{\text{d}}$) may not be appropriate for all HD patients. In addition, the rapid dialytic removal of potassium due to a high serum-dialysate potassium gradient may provoke arrhythmias and sudden death, especially with low $K_{\text{d}}$. $K_{\text{d}}$ profiling to maintain a constant serum-dialysate gradient appears to reduce ventricular arrhythmias [75]. Though the lack of automatic potassium profiling capabilities in current HD consoles limits the application of this approach, it raises further concerns about the potential harm of a low $K_{\text{d}}$ dialysate. In a multicenter prospective study, patients using $K_{\text{d}}$ of 1 mEq/L had a higher mortality compared to those receiving a 2 or 3 mEq/L [76]. In the DOPPS comparing $K_{\text{d}}$ 2 vs. 3 mEq/L, no difference in the composite outcome of all-cause mortality and arrhythmias was observed [77].

## 4. Diagnosis and Treatment of CVDs in HD Patients

There are several explanations why the diagnosis and treatment of CVDs are more complex in HD patients compared to the general population. Most landmark RCTs in the cardiovascular field exclude dialysis patients, the risk stratification scoring tools, diagnostic tools (e.g., biomarkers), as well as therapeutic agents validated in these studies, cannot be directly applied to HD patients. Patients’ symptoms, signs and laboratory measurements are, to a great extent, influenced by the HD schedule. It should also be noted that the advent and progress of CV abnormalities is a continuous procession starting long before the initiation of dialysis.

### 4.1 HF

#### 4.1.1 Diagnosis

The 2021 ESC guidelines for the diagnosis and treatment of HF consider the diagnosis of HF to include (1) symptoms and/or signs; (2) LVEF (LVEF ≤40%, LVEF 41–49% or LVEF ≥50%); and (3) for the diagnosis of HFpEF, objective evidence of cardiac structural and/or functional abnormalities consistent with the presence of LV diastolic dysfunction/raised LV filling pressures, including natriuretic peptide (B-type natriuretic peptide elevation) [8]. The New York Heart Association (NYHA) classification does not take into account the dynamic changes in volume status in dialysis patients, and that patients may have a worse NYHA class before HD than at the end of the HD session. For example, HD patients without clinically relevant cardiac structural abnormalities may exhibit typical manifestations of HF, such as nocturnal paroxysmal dyspnea and edema, due to pre-dialysis VO, which may completely disappear after appropriate dialysis and ultrafiltration. Therefore, the Acute Dialysis Quality Initiative (ADQI) Working Group XI proposed a cardiac function grading scheme dedicated to ESKD patients [78]. This HF staging schema includes the following three elements:

1⃝ Standardized echocardiographic evidence of structural and/or functional heart abnormalities;

2⃝ Dyspnea occurring in the absence of primary lung disease, including isolated pulmonary hypertension;

3⃝ Response of congestive symptoms to renal replacement treatment (RRT)/ultrafiltration.

The ADQI classification can be summarized into the following classes: Class 1—echocardiographic evidence of heart disease and asymptomatic; Class 2R—dyspnea on exertion that is relieved by RRT/ultrafiltration to NYHA class I level; Class 2NR—dyspnea on exertion that CANNOT be relieved by RRT/ultrafiltration to NYHA class I level; Class 3R—dyspnea with activities of daily life (ADL) that is relieved by RRT/ultrafiltration to NYHA class II level; Class 3NR—dyspnea with ADL that CANNOT be relieved by RRT/ultrafiltration to NYHA class II level; Class 4R—dyspnea at rest that is relieved by RRT/ultrafiltration to NYHA class III level; and Class 4NR—dyspnea at rest that CANNOT be relieved by RRT/ultrafiltration to NYHA class III level. The strength of the proposed classification is the inclusion of nonphysiological periodical fluid removal and may be useful for clinicians to differentiate patients with VO alone, and then be able to adjust the dialysis schedule (e.g., more frequent HD). However, the clinical utility and prognostic value of this HF staging classification still need to be validated in future clinical studies.

In the general population, the biomarkers BNP and N-terminal pro B-type natriuretic peptide (NT-proBNP) are important for the diagnosis of HF. However, BNP/NT-proBNP is affected by kidney function and is significantly increased in HD patients, making it challenging to establish a diagnostic cut-off value and accurately rule-in or rule-out the presence of HF. Our team found that the median NT-proBNP value is 4992 pg/mL in HD patients without HF symptoms. For HD patients with LVEF ≥60%, NT-proBNP >5741.5 pg/mL indicated VO in this population, however, it did not provide diagnostic criteria for HF [79]. Other biomarkers related to HF, such as soluble ST2 and galectin-3, though predictive of adverse cardiovascular events and/or outcomes in HD patients [80, 81], still require validation to be adopted as a diagnostic tool.

Risk stratification tools generated from the general population or the CKD population are not suitable in dialysis patients, as patients undergoing HD are faced with a very distinct spectrum of risk factors and have an increased CV risk. The real challenge is not distinguishing dark sheep from white ones, but accurately identifying darker ones. A multimarker approach that simultaneously assesses novel biomarkers with conventional biomarkers, which has been tested in patients with HF, may offer additional clinical information and improve risk stratification in the ESKD population [82]. As demonstrated in a study by Zoccali *et al*. [83], compared to traditional risk models, the combined use of CRP, BNP and ADMA increases by about one fifth the explanatory power of all-cause and CV mortality. In a prospective cohort study, the combined use of soluble ST2 (serum stimulation-2) (sST2) and NT-proBNP or hs-cTnT helped identify HD patients at higher risk [80]. This multimarker strategy is pathophysiologically reasonable and clinically promising, since profiles of multiple biomarkers reflecting different aspects of CVDs show a more comprehensive picture in ESKD. However, the benefits gained from the inclusion of over three biomarkers appear modest, and their long-term utility requires further validation.

#### 4.1.2 Treatment

The current recommended pharmacological treatment or guideline-directed medical therapy for HFrEF includes β-blockers, ARNi (angiotensin receptor/neprilysin inhibitor)/ACEI (Angiotensin Converting Enzyme Inhibitor)/ARBs (Angiotensin II receptor blockers), SGLT2i (sodium-glucose cotransporter-2 inhibitors), and mineralocorticoid receptor antagonists (MRAs). In addition, recommendations for HFpEF are made for SGLT2i, MRAs, and ARNi [84]. However, the evidence for these medications for HF in patients on dialysis is scarce and has yet to be validated [85]. A study of HD patients with HFrEF showed that ARNI reduced serum cTnT and sST2 levels and improved LVEF, supporting its safety and efficacy in the ESKD population [86].

At this time, for HD patients, optimal dialysis/ultrafiltration, including good volume control and adequate solute clearance, remains the cornerstone and the goal for the management of HF (See section 3).

### 4.2 CAD

#### 4.2.1 Diagnosis

CAD is divided into two categories, chronic coronary syndrome (CCS) and acute coronary syndrome (ACS), depending on the onset of symptoms. The early detection of CAD in HD patients is challenging, mainly because of ① High prevalence of asymptomatic CAD. Typical angina pectoris is less common in HD patients; ② Non-specific changes in baseline electrocardiography (EKG) and non-specific elevation of myocardial injury markers due to subclinical myocardial injury induced by electrolyte disturbances (especially hyperkalemia), LVH and uremic pericarditis [87]; ③ Delayed coronary angiography or coronary computed tomographic angiography due to concerns of contrast damaging kidney function in the pre-dialysis CKD G4-G5 patients.

The prevalence of asymptomatic CAD in HD patients is high, and the reasons are multifactorial: diabetic or uremic neuropathy, atypical presentation with symptoms mimicking other conditions (e.g., IDH, anemia), reduced exercise capacity. Non-invasive screening techniques can help with early detection of CAD in asymptomatic patients. Dobutamine stress echocardiography and myocardial perfusion scintigraphy are the preferred screening tools. However, clinical screening is not widely adopted, except for kidney transplant candidates [88]. The Kidney Disease Outcomes Quality Initiative recommended screening for CAD in patients with a history of revascularization, a significant reduction in left ventricular function, and a change in clinical status suggestive of a cardiac problem. Because HD/ultrafiltration has been recognized as a circulatory stressor, pre- and post-HD serial measurements of troponin T and intradialytic EKG monitoring should be considered as screening tests. Nevertheless, besides the cost-utility concerns and accessibility of screening (which varies tremendously across different regions), the challenge of deciding whether to screen asymptomatic patients is the uncertainty of the benefits of coronary revascularization, which will be discussed in the next section. For patients who are candidates for coronary revascularization, invasive testing should be considered in those with a positive stress test or with signs and/or symptoms of CAD.

#### 4.2.2 Treatment

There is limited evidence for the optimal medication strategy of CAD in patients with ESKD. In general, medication therapy focuses on three areas: antithrombotic therapy (anticoagulation/antiplatelet), lipid-lowering therapy and medications for ischemic symptoms [41]. It is important to note that certain anticoagulants are cleared by the kidneys, and their dosages need to be adjusted. For example, enoxaparin, the low molecular weight heparin (LMWH) with the most clinical evidence in ACS, is mainly cleared by the kidneys, with 40% of the total dose being cleared by the glomerulus, which requires dose reduction in case of severe renal injury and therefore is not recommended for ST-elevation myocardial infarction (STEMI) patients with CKD G5. Among statins, atorvastatin and fluvastatin are mainly metabolized in the liver via Cytochrome P450 3A4 (CYP 3A4) and excreted in bile, only <5% is excreted by the kidneys, so no dose adjustment is needed when estimated glomerular rate (eGFR) decreases. However, pravastatin, simvastatin and rosuvastatin are metabolized in the kidneys, so the dose needs to be halved for patients with CKD G3-5. It should be noted that in dialysis-dependent patients, the benefits of statin treatment are inconclusive [89, 90]. The Study of Heart and Renal Protection (SHARP) study is a placebo-controlled trial aimed to assess the efficacy of statins plus ezetimibe in patients with moderate-to-severe kidney disease, on or off dialysis. The SHARP trial showed that lowering low density lipoprotein (LDL) cholesterol with simvastatin plus ezetimibe safely reduced the risk of major atherosclerotic events in a wide range of patients with CKD. Though not powered to assess the risk reduction in dialysis-dependent patients, the benefit of statin/ezetimibe was significant in 34% of SHARP participants who began dialysis during the trial and were considered “non-dialysis” patients in the analysis [91]. Therefore, the 2013 Kidney Disease: Improving Global Outcomes (KDIGO) guideline did not recommend initiation of statins in dialysis patients. At the same time, KDIGO also suggested, in patients already receiving statin or the statin/ezetimibe combination at the time of dialysis initiation, that these agents be continued [92].

The role of coronary revascularization in CKD patients is also debated. For CCS, the ISCHEMIA-CKD trial, with 53% of the participants on dialysis and 44% on HD, found no benefit in reducing the risk of death or nonfatal myocardial infarction with an invasive strategy compared with conservative treatment [93]. For ACS, the evidence to date is limited and less robust. For STEMI, the EUDIAL Working Group supports the recommendation from the ESC guideline that the decision on immediate percutaneous coronary intervention (PCI) should be independent of the severity of kidney impairment [94]. In dialysis with non-STEMI, a large observational study suggested a potential benefit of PCI over only medical therapy [95]. When compared to coronary artery bypass graft (CABG) surgery, observational data indicated CABG is associated with higher short-term mortality, but better long-term survival for multivessel lesions [96].

Patients with CKD have a significantly higher risk of adverse clinical events following coronary revascularization compared to the non-CKD population. In particular, HD patients have a significantly increased risk of cerebrovascular events and hemorrhage. At 6 months after angioplasty in dialysis patients, recurrent ischemia was observed in 63% of patients, myocardial infarction in 23%, and death in 13% [97]. Therefore, the benefits of treatment and the potential risk of severe complications need to be weighed, and treatment decisions should be individualized.

### 4.3 Prevention of SCD

Evidence for the prevention of SCD in HD patients with antiarrhythmic drugs is inconsistent. Some studies have shown that β-blockers reduce the risk of SCD and all-cause mortality in HD patients [98, 99]. Another randomized controlled trial that included 114 dialysis patients with dilated cardiomyopathy found that carvedilol was beneficial in reducing all-cause mortality but did not significantly reduce the risk of SCD [100]. Other drugs such as ACEI/ARB, calcium channel blocker (CCB), potassium binding agents and amiodarone have not consistently been found to be effective in preventing SCD in HD patients.

Implantable cardioverter-defibrillators (ICDs) are recommended for the primary prevention of sudden death in patients with LVEF <35% and a life expectancy of more than 1 year [101]. But this evidence is mainly derived from trials excluding HD patients. Observational studies suggested that ICD implantation is associated with a reduced risk of SCD in ESKD patients with reduced LVEF (<35%) [102, 103].

However, ICD implantation in dialysis patients can also lead to undesirable complications, such as a significant increase in ESKD-related infectious complications [104].

In general, ICD implantation may reduce the incidence of lethal arrhythmias, but the benefits may be attenuated due to other causes of death. ICD-related complications and the complex comorbidities of the ESKD population make it difficult to estimate the benefit and risk of ICD implantation. Therefore, the European Dialysis Working Group did not recommend ICD implantation, and the effectiveness and applicability of ICDs for SCD prevention in the dialysis population require further study [105].

### 4.4 Treatment of Anemia And Iron Deficiency

Anemia is one of the most common complications among patients with advanced CKD. Observational studies have shown that anemia is a risk factor for the development of cardiovascular disease in dialysis patients [106, 107, 108]. Clinical treatment options other than blood transfusion were lacking until the use of ESAs. Unexpectedly, the use of ESAs to normalize hemoglobin level (>13 g/dL) may increase the risk of death and CVD, instead of improving patient prognosis [109, 110, 111]. Given this evidence, current guidelines have lowered the hemoglobin target for ESA treatment [112]. Additionally, ESA hyporesponsiveness (induced by iron deficiency, inflammation, and secondary hyperparathyroidism), rather than the hemoglobin level achieved, was associated with a higher risk of death and cardiovascular events [113].

Hypoxia-inducible factor (HIF) is a key transcription factor that senses tissue oxygen concentration and regulates physiologic responses to restore oxygen balance. HIF-α subunit, combined with the β subunit, upregulates *EPO* gene expression and iron transport in hypoxia. HIF prolyl hydroxylase inhibitors (HIF-PHIs) are orally administered small molecule compounds that stabilize HIF-α by inhibiting prolyl hydroxylase domain enzymes. HIF-PHIs increase Hb levels and total iron-binding capacity, and decrease hepcidin and ferritin levels [114]. It is hypothesized that HIF-PHIs may better mimic the physiologic process of hypoxia, improving endogenous *EPO* synthesis while diminishing exogenous *EPO* exposure, therefore resulting in a safer treatment. Several HIF-PHIs have been evaluated as oral alternatives to conventional ESA in the CKD/ESKD population [115, 116, 117, 118, 119]. Although the noninferiority design of these trials precludes any conclusions on safety issues, HIF-PHIs failed to show a better safety profile than the conventional ESAs.

Iron deficiency (ID) is common in patients undergoing HD and is a notable cause of ESA hyporesponsiveness [120]. Intravenous (IV) iron supplementation is considered the gold standard for HD patients, due to its superiority to oral iron [120]. However, given the safety concerns that IV iron, especially at high-dose, may cause oxidative stress, tissue iron deposition and increased risk of infection, the current guidelines are still inconclusive regarding the optimal management of ID in this population. The PIVOTAL trial was a RCT of 2141 patients undergoing HD randomized to high-dose proactive IV iron sucrose administration or lower-dose reactive administration. After a mean follow-up of 2.1 years, patients receiving proactive IV iron had a lower ESAs dose and transfusion rate, and more importantly, lower incidence of death, nonfatal CV events, and hospitalization, supporting a more liberal IV iron supplementation approach [121]. Interestingly, in patients with HF, ID is also a major co-morbidity. Moreover, ID is a less examined demographic of the complex co-morbidities of HF, renal impairment, and anemia [122]. RCTs in HF patients with ID, demonstrated that IV iron supplementation was safe and effective in improving functional status and exercise capacity, as well as reducing HF hospitalization [123, 124]. These findings showed that the CV benefits shown in the PIVOTAL trial were derived from physiological functions of iron beyond those of erythropoiesis.

## 5. Conclusions

HD patients have a high prevalence of CVD and mortality due to the presence of various cardiovascular risk factors. Optimized management of traditional and non-traditional risk factors may help prevent CVD and improve the prognosis of this population. Recent advances in medications for CVD are promising for ESKD patients, but their safety and efficacy need to be solidified in future well-designed clinical trials. In addition, advancements in dialysis technology may also provide new tools to treat CVD complications in the ESKD population.
